# Time‐Related Biases in Observational Studies of GLP‐1 Agonists and Cancer Risk

**DOI:** 10.1111/1753-0407.70202

**Published:** 2026-03-08

**Authors:** Fei Lu

**Affiliations:** ^1^ Department of Gastrointestinal Surgery The Fourth Affiliated Hospital of Zhejiang University School of Medicine Yiwu Zhejiang China

To the Editor,

1

We read with great interest the recent study by Cheng et al. examining the association between the use of glucagon‐like peptide‐1 receptor agonists (GLP‐1RAs) and the risk of multiple cancers among patients with type 2 diabetes [1]. Given the rapidly expanding use of GLP‐1RAs in diabetes management, evaluations of their long‐term safety profiles are of substantial clinical and public health importance. While the study addresses a timely and relevant question, several methodological aspects merit careful consideration when interpreting the reported findings.

First, the definition of GLP‐1RA exposure in the study differs from that of a conventional incident new‐user design [2, 3]. The authors classified patients based on the predominant antidiabetic therapy from baseline to the end of follow‐up, defining GLP‐1RA, insulin‐only, and metformin‐only groups while allowing limited use (≤ 6 months) of other glucose‐lowering agents during follow‐up. This approach reflects a predominant‐user design rather than initiation of therapy at cohort entry. Consequently, although follow‐up time was similar across groups, this design does not explicitly ensure that all patients were free of prior exposure to the study drugs before baseline, and the temporal alignment between treatment initiation and outcome onset may be less clearly defined. In pharmacoepidemiologic research, the absence of a pre‐baseline washout period to identify incident users may introduce prevalent user bias, as individuals already established on therapy are selectively included, potentially influencing estimated risk associations [2].

Second, the absence of a lag period is particularly relevant in the context of cancer outcomes [4]. Initiation of GLP‐1RA therapy is often accompanied by intensified clinical monitoring and increased healthcare utilization, which may increase the likelihood of detecting previously asymptomatic or indolent malignancies. Without excluding early follow‐up time, observed associations, especially those occurring within the first year of treatment, may therefore reflect detection bias rather than a causal drug effect. In this study, stratified analyses by cumulative duration of GLP‐1RA use (< 12 months vs. ≥ 12 months) suggested that elevated risks were primarily observed in shorter exposure durations, whereas longer‐term use was not consistently associated with increased risk. This pattern warrants cautious interpretation, particularly given the relatively short and comparable mean follow‐up times across treatment groups (approximately 24 months).

Notably, several recent population‐based studies have adopted design features specifically intended to address these time‐related biases [5, 6]. For example, Pasternak et al. implemented an active‐comparator new‐user design with predefined lag periods of one and two years in as‐treated analyses [5]. By explicitly excluding early outcome events, such approaches yield risk estimates that are less susceptible to reverse causation and detection bias. Differences in study design should therefore be carefully considered when comparing findings across the emerging literature on GLP‐1RA safety.

In conclusion, the study by Cheng et al. contributes valuable observational data on the association between GLP‐1RA use and cancer risk. However, its findings should be interpreted with caution, given the lack of a pre‐baseline washout period and the absence of a lag period to exclude early outcome events. Future studies employing active‐comparator new‐user designs with prespecified lag periods excluding early follow‐up would further strengthen causal inference in this important area of research.

## Author Contributions

Fei Lu – conception, manuscript draft, revision, and finalization.

## Funding

The author has nothing to report.

## Conflicts of Interest

The author declares no conflicts of interest.

## Data Availability

Data sharing not applicable to this article as no datasets were generated or analysed during the current study.
